# Retraction: PVT1 knockdown alleviates vancomycin-induced acute kidney injury by targeting miR-124 *via* inactivation of NF-κB signaling

**DOI:** 10.1039/d1ra90023d

**Published:** 2021-01-20

**Authors:** Laura Fisher

**Affiliations:** Royal Society of Chemistry Thomas Graham House, Science Park, Milton Road Cambridge CB4 0WF UK advances-rsc@rsc.org

## Abstract

Retraction of ‘PVT1 knockdown alleviates vancomycin-induced acute kidney injury by targeting miR-124 *via* inactivation of NF-κB signaling’ by Xiaoguang Zhu *et al.*, *RSC Adv.*, 2018, **8**, 31725–31734, DOI: 10.1039/C8RA05724A.

The Royal Society of Chemistry hereby wholly retracts this *RSC Advances* article due to concerns with the reliability of the data. The images in the article were screened by an image integrity expert who identified instances of image duplication and raised concerns with the integrity of the western blot panels.

In Fig. 1C, part of the image in the ‘1 day’ panel is duplicated in the ‘3 day’ panel.

In Fig. 1D, the ‘0, 1 day and 3 day’ panels show different sections of the same image.

In Fig. 2E, the si-NC panel is a direct continuation of the image shown for si-PVT1, therefore both images show the same sample.

In the western blot image in Fig. 5D (β-actin panel), the last 2 bands are mirror images of the first 2 bands, indicating that this panel was manipulated.

In the western blot panel in Fig. 7B, there is evidence of manipulation in the p-p65, p65 and β-actin panels indicating that bands may have been inserted into the figure.

The authors were asked to provide the raw data for this article, but did not respond. Given the significance of the concerns about the validity of the data, and the lack of raw data, the findings presented in this paper are not reliable.

The authors have been informed but have not responded to any correspondence regarding the retraction.

Signed: Laura Fisher, Executive Editor, *RSC Advances*

Date: 7^th^ January 2021

## Supplementary Material

